# Limitations of Out-of-Distribution Detection in 3D Medical Image Segmentation

**DOI:** 10.3390/jimaging9090191

**Published:** 2023-09-18

**Authors:** Anton Vasiliuk, Daria Frolova, Mikhail Belyaev, Boris Shirokikh

**Affiliations:** 1 Moscow Institute of Physics and Technology, Moscow 141701, Russia; vasilyuk@phystech.edu; 2 Artificial Intelligence Research Institute (AIRI), Moscow 105064, Russia; frolova@airi.net (D.F.); m.belyaev@skoltech.ru (M.B.); 3 Skolkovo Institute of Science and Technology, Moscow 121205, Russia

**Keywords:** computed tomography, magnetic resonance imaging, out-of-distribution detection, anomaly detection, segmentation

## Abstract

Deep learning models perform unreliably when the data come from a distribution different from the training one. In critical applications such as medical imaging, out-of-distribution (OOD) detection methods help to identify such data samples, preventing erroneous predictions. In this paper, we further investigate OOD detection effectiveness when applied to 3D medical image segmentation. We designed several OOD challenges representing clinically occurring cases and found that none of the methods achieved acceptable performance. Methods not dedicated to segmentation severely failed to perform in the designed setups; the best mean false-positive rate at a 95% true-positive rate (FPR) was 0.59. Segmentation-dedicated methods still achieved suboptimal performance, with the best mean FPR being 0.31 (lower is better). To indicate this suboptimality, we developed a simple method called Intensity Histogram Features (IHF), which performed comparably or better in the same challenges, with a mean FPR of 0.25. Our findings highlight the limitations of the existing OOD detection methods with 3D medical images and present a promising avenue for improving them. To facilitate research in this area, we release the designed challenges as a publicly available benchmark and formulate practical criteria to test the generalization of OOD detection beyond the suggested benchmark. We also propose IHF as a solid baseline to contest emerging methods.

## 1. Introduction

In recent years, deep learning (DL) methods have achieved human-level performance in automated medical image processing. However, the development of these methods on a large scale is slowed by several factors. One such factor is the unreliable performance of DL models when the data come from a distribution different from the training one [1]. These differences are common in medical imaging, population, demographic, or acquisition parameter changes or new imaging modalities.

Out-of-distribution (OOD) detection helps to identify the data samples with such differences, hence increasing the reliability and safety of a DL model. For instance, detected cases could be marked as rejected, preserving the model’s performance, or reported to the experts, preventing the model from failing silently. The ability to report or reject unreliable cases is now considered a necessary capability to enable safe clinical deployment [2].

OOD detection with natural images is a well researched area [3] where several established benchmarks [4,5] facilitate its development. Moreover, these methods directly scale to 2D medical images, resulting in multiple algorithms [6,7,8] and also a benchmark [9]. At the same time, OOD detection with 3D medical images remains poorly explored, although 3D medical image segmentation is one of the most addressed tasks in medical imaging [10] with outstanding practical usefulness, e.g., quantifying anatomical structures, pathologies, or important biomarkers.

The primary cause of this poor exploration is the lack of datasets and benchmarks with a correct problem design. For example, one party may use private data [11], while another simulates synthetic anomalies that are unlikely to occur in clinical settings [12]. A study can be limited to a single distribution shift (e.g., changes in the scanning location [11]), thus lacking the diversity of setups. Also, studies may be restricted to uncertainty estimation [13] or anomaly detection [12] methods, leaving the full spectrum of approaches uncovered. Such issues limit a fair comparison of the proposed approaches.

In this paper, we investigate the effectiveness of OOD detection when applied to 3D medical image segmentation, closing the outlined gaps in prior work. To enable a correct comparison, we designed a *diverse* set of challenges using *publicly available data* with a *downstream segmentation task* and simulation of *clinically occurring anomaly sources*. Besides the problem design, such a study requires appropriately selected state-of-the-art methods. We note that several areas (e.g., anomaly detection and uncertainty estimation) share motivation and methodology with OOD detection. Therefore, we review all related areas and, in contrast to the previous works, present complete methodological coverage.

An extensive evaluation of the six selected methods resulted in our main conclusion: state-of-the-art OOD detection falls short of achieving optimal performance with 3D medical images. We show that the methods not designed for segmentation completely failed in most setups, scoring from 0.84 to 0.59 for the false-positive rate (FPR) on average, which was not far below the 0.95 FPR of the random guessing (a lower FPR is better). Two methods specifically designed for 3D segmentation achieved 0.38 and 0.31 mean FPRs, further reducing the error by about two times. At the same time, we show that these errors can be reduced even further with a simple approach.

We show this space for improvement by developing a histogram-based method called Intensity Histogram Features (IHF). IHF achieved comparable and often superior results to its competitors, with a 0.25 mean FPR. It also scored 0 for the FPR in multiple challenges, indicating that the distribution shifts in 3D medical imaging can often be detected using image intensity histograms, while the DL-based methods overlook this domain feature. Therefore, we consider current DL-based OOD detection to be far from unveiling its full potential and assume it can be further improved.

Given IHF’s negligible computational costs compared to DL, we suggest it as a baseline to contest the emerging OOD detection methods. Furthermore, we propose using the designed challenges as a benchmark for developing new methods. Correct problem setting; in-depth analysis with simple methods, such as IHF; and ablation studies with synthetic data confirm that our benchmark makes it possible to estimate the quality of implementing general OOD detection instead of classifying a priori-known anomaly types. Thus, summarizing our contributions, we outline the following:

1. We demonstrate the severe limitations of the existing OOD detection methods with 3D medical images;
2. We designed and now release the corresponding benchmark that can be used as a starting point for related research;
3. We propose a method, IHF, and suggest it as a solid baseline for OOD detection with 3D medical images.

Below, we describe the data used in our study and the problem setup (Section 2). Then, we review and select state-of-the-art and core methods from related fields and also detail IHF (Section 3). Finally, we present the results (Section 5) and discuss the limitations and implications of our study (Section 6).

## 2. Data

In contrast to the fields of 2D natural and medical images, no established OOD detection benchmark with a correct problem setting exists for 3D medical images. For example, Karimi et al.  [11] used a variety of brain and abdominal CT and MRI datasets but included private ones. The authors also studied only a single distribution shift, changes in the scanning location, which did not allow the estimation of the general performance. Zimmerer et al. [12] created an OOD detection challenge by simulating synthetic anomalies in brain MR and abdominal CT images. However, their setup lacks a downstream task (e.g., segmentation), so the study is limited to unsupervised anomaly detection methods. The synthesis of local corruptions, as in [12], can also lead to evaluation biases, which we show with our analysis. On the other hand, Lambert et al. [13] included datasets with a segmentation task but limited the considered methods to supervised uncertainty estimation.

Given the disagreement of setups, partial problem coverage, or privacy, we designed the OOD detection challenges from scratch following three core principles:

- We included two large *publicly available* CT and MRI in-distribution (ID) datasets to cover the most frequent volumetric modalities.
- We ensured both datasets had *a downstream segmentation task*, allowing us to use the full spectrum of methods.
- We selected *diverse* OOD datasets that simulated the *clinically occurring sources of anomalies*: changes in acquisition protocol, patient population, or anatomical region. All these datasets are publicly available.

We also synthesized several medical imaging artifacts as anomaly sources. Generating synthetic anomalies is a popular approach, applied for 3D images [12,13] as well as 2D images [4,5]; this approach also allowed us to conduct controlled ablation studies at different distortion levels.

We made the resulting benchmark publicly available (https://github.com/francisso/OOD-benchmark, accessed on 6 August 2023). All related CT and MRI datasets are detailed in Section 2.1 and Section 2.2. The problem setting is described in Section 2.3.

### 2.1. Lung Nodules Segmentation

**Figure 1:**
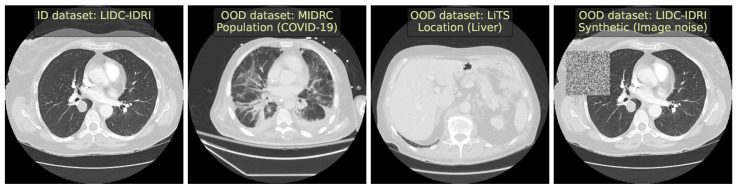
Examples of CT images (representative axial slices) from different simulated OOD sources in our benchmark.

We constructed a total of six challenges on CT data, including two synthetic ones. We give visual examples of data samples in Figure 1 and detail the ID dataset and every setup below. The segmentation quality of the downstream model is provided in Table 1.

**ID dataset.** As an ID dataset, we used LIDC-IDRI [14]. It contains 1018 chest CT images with the lung nodules segmentation task. We removed cases without nodules since they do not contribute to training a segmentation model. Then, we randomly split the remaining 883 images 4:1 into the train and test, stratified by the number of nodules.

**OOD source:** ***scanner.*** To simulate a covariate shift, we selected Cancer500 [15] which has the same downstream task as the ID dataset but is obtained with different scanners and acquisition protocols. It contains 979 chest CT images. We excluded images with a low resolution (less than 64 axial slices) and no annotated nodules, resulting in 841 images left.

**OOD source:** ***population.*** To simulate a patient population shift, we used two datasets with similar semantic content but a different downstream task. These datasets are Medseg9 (https://radiopaedia.org/articles/covid-19-3, accessed on 6 August 2023) and MIDRC [16], containing 9 and 154 chest CT images, respectively, with COVID-19 cases. Excluding all non-COVID-19 cases, the merged dataset has 120 images.

**OOD source:** ***location (liver).*** To simulate a semantic shift, we selected a dataset of the same modality but a different body region. Here, we used LiTS [17], a dataset with 201 abdominal CT images.

**OOD source:** ***location (head).*** Similarly, we included CT-ICH [18], a dataset with 75 head CT images.

**OOD source:** ***synthetic (image noise).*** We simulated local image corruptions by applying damaging transformations to the test cases of the ID dataset. The transformations include blurring, changing image contrast, or inserting Gaussian noise in a randomly selected image crop.

**OOD source:** ***synthetic (elastic).*** We simulated tissue anomalies by applying an elastic transform of random severity.

### 2.2. Vestibular Schwannoma Segmentation

**Figure 2:**
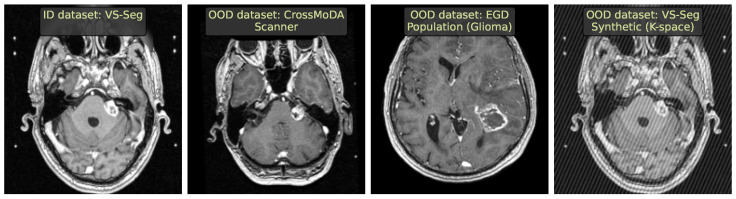
Examples of MRI images (representative axial slices) from different simulated OOD sources in our benchmark.

We constructed a total of seven challenges on MRI data, including four synthetic ones. We give visual examples of data samples in Figure 2 and detail every setup below. The segmentation quality of the downstream model is provided in Table 2.

**ID dataset.** As an ID dataset, we used VS-Seg [19]. It contains 242 brain T1c MRIs with the vestibular schwannoma segmentation task. We removed cases with empty target masks and split the remaining 239 images 2:1 into the train and test.

**OOD source:** ***scanner.*** To simulate a covariate shift, we selected data with the same semantic content and downstream task but obtained using different scanners and acquisition protocols. Here, we chose CrossMoDA ETZ as a subset of the CrossMoDA 2022 Challenge dataset [20] with 105 brain T1c MR images and used it without changes.

**OOD source:** ***population (glioblastoma).*** To simulate a patient population shift, we used EGD [21], a dataset with 774 brain MRIs of four modalities (FLAIR, T1, T1c, T2) with a glioma segmentation task. We reduced covariate shift by using only the T1c modality from the Siemens Avanto 1.5T scanner, as in VS-Seg, resulting in 262 selected images.

**OOD source:** ***population (healthy).*** Additionally, we simulated a patient population shift using healthy cases instead of changing the pathology. We used the CC359 [22] dataset with 359 brain MR images of the T1 modality. We note, however, that CC359 images differ in their vendor and scanning protocol and do not contain contrast enhancement, so this setup has a secondary OOD source, a covariate shift.

**OOD source:** ***synthetic (K-space noise).*** We synthesized MR imaging artifacts, known as Herringbone artifacts, at different magnitudes. This resulted in visible spikes across the whole image due to anomaly points in the K-space.

**OOD source:** ***synthetic (anisotropy).*** We synthesized the wrong resolution by downsampling the image and upsampling it back along one randomly chosen axis.

**OOD source:** ***synthetic (motion).*** We synthesized two types of MR imaging artifacts that can happen due to the patient’s motion. One is ghosting, which appears as shifted copies of the original image; the other exploits RandomMotion simulation from the torchIO library [23].

**OOD source:** ***synthetic (image noise).*** The same pipeline is used as for CT images.

### 2.3. Problem Setting

We define the OOD detection problem as a classification between samples from a source distribution (ID) and abnormal samples from a novel different distribution (OOD). The core assumption is that the abnormal sample distribution is unknown and cannot be computed in advance. Thus, we approximate the anomaly distribution by constructing diverse setups representing clinically occurring cases. Consequently, a reliable method must be generalized to novel sources of anomalies besides attaining the desired accuracy on the suggested test set.

Providing a downstream segmentation task, we removed any constraints on the method design. One can use the model’s features, uncertainty estimates, or an auxiliary model to detect outliers. In all cases, a method should output a single number called the *OOD score* for every testing image; a higher score means a higher outlier likelihood.

## 3. Methods

### 3.1. Methods Selection

Several sub-topics, including anomaly detection (AD), novelty detection, uncertainty estimation (UE) and outlier detection, share motivation and methodology with OOD detection. Despite subtle differences between these topics, the approaches are similar and most of them can be applied for OOD detection with minimal changes, as shown in [24]. So, we followed the structure of [24] and selected core methods from OOD detection, UE and AD. In our selection, we prioritized methods already implemented for medical imaging (e.g., in [11,25,26]).

As a universal baseline, the maximum probability of a softmax output can be used to detect OOD samples without any model modifications [4]. In practice, however, the entropy of the softmax output (**Entropy**) is used instead [11,25,27]. We consider Entropy a starting point for all other approaches and show its performance in our benchmark.

The softmax entropy captures the total uncertainty, while the OOD measure corresponds only to the epistemic uncertainty, as explained in [28]. Thereby, one can use epistemic uncertainty estimation techniques to improve Entropy. Among the others, Deep **Ensemble** [29] is considered the state-of-the-art approach for UE. To use Ensemble, one computes mutual information or variance over several predictions for a single image to obtain an epistemic uncertainty map. An alternative way to obtain multiple predictions is Monte Carlo dropout (**MCD**) [30], which we also included in our comparison.

Further, we included the approach of [11], which directly addresses OOD detection on 3D medical images. The authors applied singular value decomposition (**SVD**) to the network features and used the singular values as image embeddings. The OOD score is then the distance from a sample’s embedding to its nearest neighbor in the training set.

Better uncertainty estimates can be obtained by modifying the downstream model, although such modifications can harm the model’s performance. We included one popular modification, generalized ODIN (**G-ODIN**) [31], in our study. Finally, one can use an auxiliary model dedicated solely to anomaly detection. Such AD methods were extensively compared in the Medical Out-of-Distribution (MOOD) challenge [12]. We implemented the best solution from MOOD 2022 and included it in our experiments as **MOOD-1**.

Discussing the auxiliary AD models, we intentionally excluded the reconstruction-based methods (e.g., auto-encoders, generative-adversarial nets) from our consideration. Firstly, these methods performed substantially worse in MOOD 2022 than self-supervised learning-based ones (e.g., MOOD-1) [26]. Liang et al. [32] also demonstrated them to score far behind self-supervised learning. Moreover, Meissen et al. [33] highlighted the severe limitations of auto-encoders applied to OOD detection in a similar setup. Given this critique, we do not include the reconstruction-based approaches in our experiments.

So, we consider the following methods: Entropy, Ensemble, MCD, SVD, G-ODIN and MOOD-1. Since some of them are designed for the downstream classification task, we detail their adaptation to segmentation below.

### 3.2. Method Implementation

To preserve a fair comparison, we added only trivial and unavoidable modifications. We also tested (in preliminary experiments) any additional component or a critical hyperparameter of every method and selected the best-performing setting.

#### 3.2.1. Entropy

Our downstream task is binary segmentation, where the sigmoid function is applied to the network’s outputs. We note that two-classes softmax can be derived from the sigmoid. Then, Entropy follows the implementation from [11,27], computing the average entropy value over the predicted area (i.e., positive class). We set the OOD score to 0 in the case of the empty predicted mask.

#### 3.2.2. Ensemble

We trained 5 U-Net models with different initializations and calculated the uncertainty map as the voxel-wise standard deviation of the five corresponding predictions. The OOD score is the average of this uncertainty map.

#### 3.2.3. MCD

We implemented MCD by introducing a dropout layer before every down- and up-sampling U-Net layer. Then, we calculated voxel-wise standard deviations of 5 inference steps with a dropout rate of 0.1. The OOD score is the average of the resulting uncertainty map.

#### 3.2.4. SVD

We followed [11] without any changes.

#### 3.2.5. G-ODIN

We preserved the original structure of the G-ODIN output layer [31]; the only difference was that we substituted the linear layers with the convolution ones. These convolution layers had kernels of size $1^{3}$, so the procedure remained equal to the classification of every voxel. Then, we used the best-reported G-ODIN DeConf-C${}^{*}$ variant to calculate uncertainty.

#### 3.2.6. MOOD-1

The best-performing MOOD solutions are trained to segment synthetically generated anomalies in a self-supervised fashion [26]. So, our MOOD-1 implementation is based on this cut-paste-segment approach, which won MOOD 2021 [34]. We then supplemented it with technical improvements from 2022’s best solution (team CitAI), such as one-cycle learning and ensembling over five models. The subject-level OOD score is calculated as the mean of the top 100 anomaly probabilities.

#### 3.2.7. Volume Predictor

To demonstrate that some semantic differences might be trivial from the model’s perspective but not captured by other methods, we used the total volume of a prediction (positive class) as an OOD score. Since a predicted volume can vary in any direction, we considered the sample an outlier if the volume was below $\frac{q}{2}$-th or above the $100-\frac{q}{2}$-th percentile of the ID, thus retaining 100−q TPR.

### 3.3. Intensity Histogram Features

We propose an unsupervised method based on image intensity histograms as embeddings to contest the DL algorithms. Our design is motivated by two other works. Karimi et al. [11] showed that SVD can efficiently reduce full-image-sized network features. We note a space for improvement in their method: one can optimize the choice of the network’s layer to apply SVD. Zakazov et al. [35] suggested that the earlier network’s layers contain the most domain-specific information. Following the latter suggestion, we hypothesize that we can extract enough domain-specific information directly from the image (i.e., the zeroth network’s layer). A histogram is a convenient way to do so.

**Figure 3:**
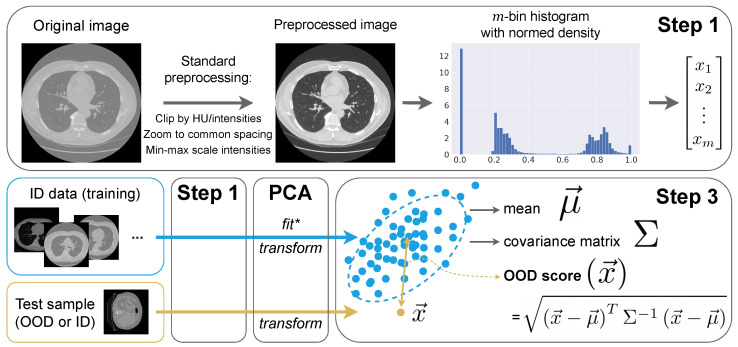
The proposed OOD detection method, called *Intensity Histogram Features (IHF)*. It consists of three steps: calculating a *m*-dimensional vector as histogram bin values from the preprocessed image (*Step 1*), fitting and applying *PCA* to the occuring data, and calculating the Mahalanobis distance between a test vector and ID sample distribution (*Step 3*). We apply IHF to the 3D images and illustrate the process using 2D axial slices for simplicity. (* PCA is fitted once on all training data.)

We schematically present our method, called Intensity Histogram Features (IHF), in Figure 3. It consists of three steps: (1) calculating intensity histograms of images and using them as vectors, (2) reducing their dimensionality with PCA and (3) running an outlier detection algorithm on these vectors.

Step 1: Preprocessing and histograms

All images undergo the same preprocessing pipeline to standardize the intensity distribution:

1. We interpolate images to the median ID spacing. So, in all CT and MRI experiments, we use 1×1×1.5 mm.
2. We clip image intensities to [−1350,300] Hounsfield units for CT (a standard lung window) and [1th percentile, 99th percentile] for MRI.
3. We MinMax-scale image intensities to the [0,1] range.

Given a preprocessed image *x*, we compute a probability density function of its intensities in *m* bins, a histogram $e\left(x\right)\in R^{m}$ and further use these vectors e(x).

Step 2: Principal Component Analysis (PCA)

As an optional step, we use PCA to reduce the dimensions *m*. The main reason to use it is that some outlier detection algorithms at *Step 3* behave unstably in high dimensional spaces. For instance, calculating Mahalanobis distance requires reversing the empirical sample covariance matrix and this matrix is likely to become ill-conditioned or singular with a larger *m*.

Therefore, we fit PCA${}_{v}$ once on the training data $E_{tr}$ to preserve v=99.99% of the explained variance. This way, we eliminate the potential instability and preserve the distribution properties. $E_{tr}$ consists of row-vectors $e(x_{tr})$ for all training images $x_{tr}\in X_{tr}$. Further, we use transformed vectors $\tilde{e}\left(x\right)={\mathrm{PCA}}_{v}\left(e\left(x\right)\right)$.

Step 3: OOD detection algorithm

To calculate an OOD score for *x*, we can apply any distance- or density-based outlier detection method. As in [36], we can calculate the Mahalanobis distance $S_{Mah}\left(x\right)$:

(1) $$S_{Mah}\left(x\right)=\sqrt{{\left(\tilde{e}\left(x\right)-\hat{\mu }\right)}^{T}{\hat{\Sigma }}^{-1}\left(\tilde{e}\left(x\right)-\hat{\mu }\right)},$$

where $\hat{\mu }$ and $\hat{\Sigma }$ are the estimated mean and covariance matrix on the training set, $\hat{\mu }=\frac{1}{|X_{tr}|}\sum_{x_{tr}\in X_{tr}}\tilde{e}\left(x_{tr}\right)$ and $\hat{\Sigma }=\frac{1}{|X_{tr}|}\sum_{x_{tr}\in X_{tr}}\left(\tilde{e}\left(x_{tr}\right)-\hat{\mu }\right){\left(\tilde{e}\left(x_{tr}\right)-\hat{\mu }\right)}^{T}$. Alternatively, one can calculate the distance to the nearest neighbor (min-distance) $S_{NN}\left(x\right)$, as in [11]:

(2) $$S_{NN}\left(x\right)=\min_{x_{tr}\in X_{tr}}\left|\right|\tilde{e}\left(x\right)-\tilde{e}\left(x_{tr}\right){\left|\right|}_{2}.$$

Using $S_{Mah}$ (Equation (1)) and $S_{NN}$ (Equation (2)) corresponds to the methods IHF-Mah and IHF-NN, respectively. We included them in our experiments independently.

## 4. Experimental Setup

### 4.1. Downstream Task

We have 3D CT and MRI datasets with a segmentation task. So, we adhere to the standard approaches to train a segmentation model in all methods that require the latter.

#### 4.1.1. Data Preprocessing

We describe preprocessing in IHF, Step 1 (Section 3.3); it is the same in all experiments.

#### 4.1.2. Architecture and Training

In all experiments, we use 3D U-Net [37], a standard architecture for segmentation. We train it on patches of 64 axial slices, with a batch size of 3, Adam optimizer and a learning rate of $10^{-4}$ for 30 epochs, 1000 iterations each. In a batch, patches from different images are padded if necessary. We minimize the sum of Binary Cross-Entropy and Focal Tversky losses [38] to achieve high segmentation sensitivity.

#### 4.1.3. Segmentation Evaluation

We train all models on the training part of ID datasets. Then, we can evaluate their segmentation quality on the corresponding testing part of the OOD datasets, showing its possible decline. These segmentation results are given in Table 1 for the CT and Table 2 for the MRI datasets.

### 4.2. OOD Detection Evaluation

Given the ID test data, we measure the OOD detection quality against it for all the suggested OOD setups, similarly to the classification task. Outliers occur rarely in practice, so we aim to measure detection quality when most ID samples are being preserved with respect to relatively rare OOD events. In this case, one of the most convenient classification metrics is the false-positive rate at a 95% true-positive rate (FPR), so we report FPR as our primary metric in Table 3. We also show AUROC in Table 4 for consistency with other studies.

## 5. Results

In this section, we report on our experiments and results. We start by benchmarking all considered methods, then present the analysis of the benchmark design and conclude with the ablation study on synthetic data.

### 5.1. Benchmarking

Table 3 presents the primary results of our study. Uncertainty-based methods, not designed for segmentation, mostly failed in the suggested setups. Entropy, Ensemble, MCD and G-ODIN gave substantially higher FPR than the other methods, with only G-ODIN slightly surpassing a simple *Volume* predictor. Methods dedicated to segmentation performed better on average. For instance, MOOD-1 achieved 0.36 and 0.41 average FPR on CT and MRI data, respectively. SVD improved further; it appeared to be the only reliable studied method, providing 0.42 and 0.21 mean FPR.

Then, we contested SVD performance by the proposed IHF. In combination with min-distance, IHF-NN provided the best average scores across the studied challenges, 0.43 and 0.08 FPR, respectively. In combination with Mahalanobis distance, IHF-Mah provided practically worse results in the CT setups. Although IHF-Mah is not the best version, it was historically the first and we submitted it to MOOD 2022 (m=150, no PCA). We placed second as team AIRI (http://medicalood.dkfz.de/web/, accessed on 6 August 2023) with the earliest IHF version, supporting its robustness by the independent evaluation.

**Figure 4:**
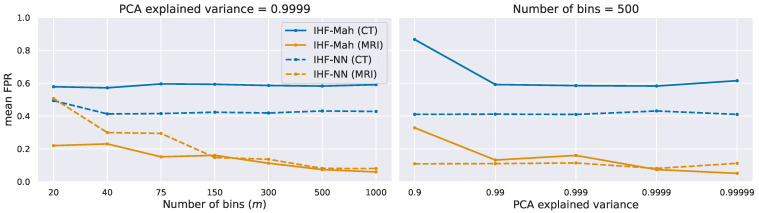
Dependence of IHF on its two hyperparameters: the number of histogram bins (*m*) and explained variance in PCA (*v*). We give the results for both IHF variants and CT and MRI setups.

We also conducted an ablation study to verify IHF robustness. As shown in Figure 4, we tested IHF performance by varying its two parameters, the number of bins (*m*) and explained variance ratio (*v*). Our findings indicated a consistent behavior regardless of the parameter choice, with a slight trend of improved quality at a larger *m*.

Both IHF variants performed comparably or better on average than SVD and, consequently, the other studied methods. Therefore, we conclude that the histograms of image intensities are descriptive enough to detect most of the suggested OOD cases. At the same time, neural networks might omit important domain features in this problem. We thus hypothesize that neural network-based OOD detection can be further improved and leave this promising direction for future research.

We present the same comparison in terms of AUROC in Table 4. Although AUROC is not our primary metric, it roughly preserves the same relative ranking as the studied methods, not contradicting our main message.

### 5.2. In-Depth Benchmark Analysis

Further, we emphasize the significance of constructing a *correct* benchmark to study the methods. An analysis of our experimental results suggests the following:

- OOD methods should be studied under a benchmark with diverse OOD challenges.
- Setups should represent clinically occurring cases.
- Potential biases in the benchmark should be explored using simple methods, such as IHF or Volume predictor.

It is often possible to develop a method tailored to specific OOD sources where it thrives but fails in the other setups. For example, G-ODIN demonstrated near-perfect results in the Population and Scanner setups on MRI data but yielded the worst scores in the others. In practice, however, the precise anomaly source is always unknown and a general OOD detector with an acceptable average performance is needed. The true method effectiveness can be estimated only in the context of diverse setups.

Secondly, OOD sources should accurately represent or simulate the clinically occurring cases. For instance, the Synthetic (Noise) setup, as introduced in [26] and reproduced in our study, is not supported by any medical imaging process. MOOD-1 achieved the highest performance in this setup because its training objective is closely aligned with the anomaly synthesis process. However, performing well in this and similar cases is of no clinical value and, consequently, biases the methods’ evaluation towards explicitly unrealistic scenarios.

Finally, our analysis revealed that OOD challenges might contain implicit but trivial features. If a benchmark focuses solely on any such feature, we can design a method that exploits this feature, leading to deceptive conclusions about the generalized performance. Instead, we suggest using simple methods to reveal biased features beforehand. For example, near-perfect IHF results in several setups demonstrated that certain anomalies are actually trivial intensity changes, reinforcing the need to design diverse benchmarks.

To ensure the methods’ generalization, we calculate the Fechner correlation between their results and the results of the *simple methods*. We show that, apart from SVD, the others exhibit a weak correlation with the Volume or IHF scores (Table 5). So, the examined methods mostly do not rely on trivial features, such as image intensity distribution. However, SVD showed a correlation of 0.54 with the Volume scores, suggesting its hindered generalization on new data sources with a small difference in the predicted area volume.

### 5.3. Ablation Study on Synthetic Data

**Figure 5:**
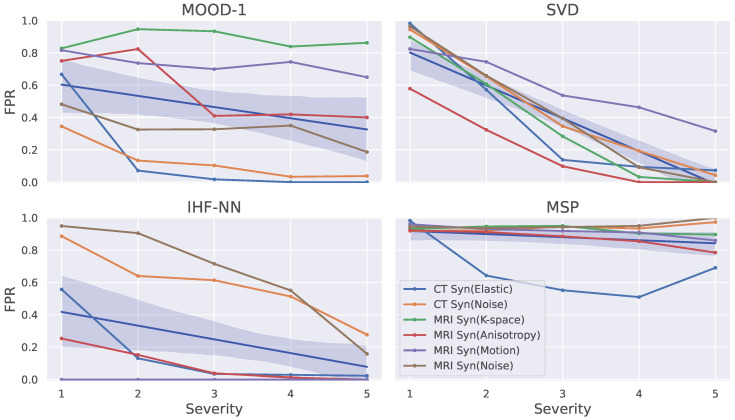
FPR under synthetically distorted data for every distortion severity level. Blue line indicates method’s average trend across presented challenges with 95% confidence interval. The other UE methods (MCD, Ensemble and G-ODIN) are excluded since their average trend is similar to Entropy.

We show the OOD detection results on synthetic data for different distortion levels in Figure 5. Distortion levels were chosen perceptually from one (barely noticeable distortion) to five (heavily distorted image). The general trend is that more distorted images are easier to detect. Here, SVD exhibited the steepest average slope and behaved almost linearly with the increasing severity level, suggesting that we have considered challenging but solvable tasks.

Different methods exhibited different sensitivities to the level of distortion required for detection. Entropy and the other UE methods started to operate effectively only at level 3, while IHF detected anomalies at a minimal level. So, we conclude that the methods should be studied across a wide range of anomaly severity levels. Additionally, we show that MOOD-1 depends more on the OOD source than the severity level: it failed in Motion and K-space setups while almost perfectly detecting Noise and Elastic deformations independently of the severity level. Moreover, MOOD-1 and IHF behaved inversely to each other in the Noise and Elastic setups. Such diverse behavior suggests the need to study methods across a wide range of anomaly types.

## 6. Discussion

Besides benchmarking the OOD detection methods, our study also suggested practical ideas for building a correct benchmark. We mainly highlight the data diversity in multiple dimensions and the clinical relevance of the setups. However, we leave several critical questions undeveloped, thus opening the corresponding research directions, which we discuss below.

### 6.1. Benchmark Design

We selected the datasets that represent the clinically occurring sources of OOD data. We confirmed the importance of the constructed challenges by the degraded downstream performance (Table 1 and Table 2). However, we cannot certainly state that the highlighted sources are the only differences between the distributions. While we name the primary difference in each case (e.g., acquisition parameters, patient populations), the distributions might differ due to several causes and others exist outside of our consideration. So, a more refined benchmark development with the controlled distinction between the OOD sources would greatly facilitate further research in this area.

Furthermore, the question of whether any semantic difference should be considered abnormal requires additional investigation. For instance, the Population (Healthy) setup is considered OOD due to this apparent semantic difference—healthy cases instead of pathological ones. Segmentation models often yield correct empty predictions for such images regardless of any OOD detection decision. In this case, rejecting a correct prediction should be considered a false-negative instead of a true-positive outcome, lowering the false-positive rate.

### 6.2. Uncertainty Estimation

Our study verifies that the epistemic uncertainty is better suited for OOD detection than the total uncertainty, since MCD and Ensemble achieved better results than Entropy. Nevertheless, the question of how to aggregate the uncertainty map into a single score remains open. On the one hand, aggregating uncertainty over the predicted volume offers certain advantages compared to the whole image aggregation, especially when dealing with 3D images, where the area of interest may occupy only a small portion of the entire image. While this aggregation showed improved results for the Entropy method, it cannot rank samples with an empty predicted mask and does not trigger anomalies outside of the predicted area. Contrarily, aggregating uncertainty as a simple average over the whole image provided better results for MCD and Ensemble. Developing a reasonable UE method for 3D images is thereby a possible direction for future research.

### 6.3. Other IHF Applications

Additionally, we explored alternative applications of the proposed IHF method. We noted its strong performance in medical imaging tasks such as contrast detection and domain classification. In this paper, however, we do not delve into the potential IHF applications, as they lie beyond the scope of the OOD detection problem.

## 7. Conclusions

In this paper, we have conducted an extensive investigation of OOD detection on 3D medical images. Our results revealed that the established approaches, including uncertainty estimation and anomaly detection, do not provide a reliable performance. These methods predicted an unacceptably high number of false positives (0.31 mean FPR at best) and failed to generalize. We also showed that they possess space for improvement. To achieve this, we developed a histogram-based method, IHF, which achieved comparable and often superior results to its competitors. Thereby, we indicated that the distribution shifts in 3D medical imaging can often be detected using intensity histograms, while the DL algorithms neglect this domain feature. Although IHF achieved better average results, its performance was surpassed in multiple challenges, emphasizing the need and possibility for developing a robust and general OOD detection method.

We constructed and released the corresponding challenges as a benchmark for OOD detection on 3D medical images, proposing IHF as a solid baseline to contest new methods.

## Figures and Tables

**Table 1:** Segmentation quality in the considered CT setups. We provide the Dice score (DSC, higher is better (↑)) and average number of false-positive detections per image (avg. FP, lower is better (↓)) as our metrics.

Metric	ID Dataset	OOD Challenges					
	LIDC	Loc (Head)	Loc (Liver)	Scanner	Pop (COVID-19)	Syn (Noise)	Syn (Elastic)
DSC (↑)	0.50±0.27	0.21±0.41	0.03±0.16	0.18±0.24	0.00±0.00	0.32±0.30	0.13±0.23
Avg. FP (↓)	5.2±6.9	2.6±4.6	10±16	6±15	13±10	12±25	5.4±4.5

**Table 2:** Segmentation quality in the considered MRI setups in terms of Dice score (DSC, higher is better (↑)).

Metric	ID Dataset	OOD Challenges						
	VS-Seg	Pop (Healthy)	Pop (Glioma)	Scanner	Syn (K-Space)	Syn (Anisotropy)	Syn (Motion)	Syn (Noise)
DSC (↑)	0.92±0.05	0.85±0.36	0.86±0.34	0.89±0.12	0.83±0.023	0.87±0.01	0.91±0.06	0.57±0.41

**Table 3:** Comparison of the considered OOD detection methods in terms of FPR@TPR95% scores (lower is better). We highlighted the best scores in every row in **bold** and ranked the methods by their average performance. The first and second sections correspond to CT and MRI setups, respectively.

OOD Setup	IHF-NN	SVD	IHF-Mah	MOOD-1	G-ODIN	Volume	MCD	Ensemble	Entropy
Location (Head)	**0.00**	**0.00**	**0.00**	0.12	0.55	0.53	0.36	0.51	0.56
Location (Liver)	0.51	**0.13**	0.64	0.56	0.56	0.84	0.89	0.93	0.78
Population (COVID-19)	0.54	0.75	0.72	**0.51**	0.54	0.82	0.58	0.58	0.87
Scanner	0.88	0.89	0.85	**0.73**	0.92	0.86	0.89	0.90	0.83
Synthetic (Elastic)	**0.15**	0.37	0.67	0.16	0.59	0.81	0.42	0.37	0.84
Synthetic (Image noise)	0.49	0.37	0.62	**0.11**	0.89	0.85	0.87	0.82	0.81
Population (Glioblastoma)	**0.00**	**0.00**	**0.00**	0.10	0.21	0.01	0.85	0.81	0.86
Population (Healthy)	**0.00**	**0.00**	**0.00**	0.11	**0.00**	**0.00**	0.88	10.0	0.85
Scanner	**0.00**	**0.00**	**0.00**	0.15	**0.00**	0.74	0.63	0.66	0.89
Synthetic (K-space noise)	**0.00**	0.36	**0.00**	0.88	0.88	0.90	0.82	0.77	0.73
Synthetic (Anisotropy)	0.09	0.20	**0.05**	0.57	0.88	0.93	0.77	0.77	0.81
Synthetic (Motion)	**0.00**	0.58	**0.00**	0.73	0.93	0.94	0.85	0.88	0.91
Synthetic (Image noise)	0.47	0.33	0.47	**0.30**	0.56	0.71	0.78	0.75	0.75
CT average	0.43	0.42	0.58	**0.36**	0.67	0.79	0.67	0.68	0.78
MRI average	0.08	0.21	**0.07**	0.41	0.50	0.60	0.80	0.81	0.83

**Table 4:** Comparison of OOD detection methods in terms of AUROC scores (higher is better). We highlighted the best scores in every row in **bold** and ranked the methods by their average performance. The first and second sections correspond to the CT and MRI setups, respectively.

OOD Setup	IHF-NN	IHF-Mah	SVD	G-ODIN	Volume	MOOD-1	MCD	Ensemble	Entropy
Location (Head)	**1.0**	**1.0**	**1.0**	0.83	0.73	0.83	0.85	0.79	0.62
Location (Liver)	0.89	0.85	**0.97**	0.88	0.65	0.61	0.42	0.45	0.67
Population (COVID-19)	**0.88**	0.83	0.74	0.86	0.76	0.66	0.79	0.80	0.72
Scanner	**0.73**	**0.73**	0.58	0.72	0.68	0.51	0.58	0.55	0.65
Synthetic (Elastic)	**0.97**	0.83	0.86	0.85	0.77	0.78	0.84	0.85	0.65
Synthetic (Image noise)	0.81	0.77	**0.84**	0.75	0.67	0.80	0.56	0.61	0.59
Population (Glioblastoma)	**1.0**	**1.0**	**1.0**	0.96	0.68	0.87	0.44	0.41	0.14
Population (Healthy)	**1.0**	**1.0**	**1.0**	**1.0**	0.68	0.86	0.44	0.16	0.15
Scanner	**1.0**	**1.0**	**1.0**	**1.0**	0.77	0.83	0.70	0.74	0.59
Synthetic (K-space noise)	**1.0**	**1.0**	0.86	0.81	0.66	0.24	0.56	0.63	0.66
Synthetic (Anisotropy)	**0.98**	**0.98**	0.94	0.81	0.68	0.57	0.63	0.63	0.71
Synthetic (Motion)	0.99	**1.0**	0.75	0.78	0.68	0.48	0.57	0.54	0.57
Synthetic (Image noise)	0.81	0.83	0.85	**0.88**	0.66	0.77	0.58	0.58	0.56
CT average	**0.88**	0.84	0.83	0.82	0.71	0.70	0.67	0.68	0.65
MRI average	**0.97**	**0.97**	0.92	0.89	0.69	0.66	0.56	0.53	0.48

**Table 5:** Fechner correlation coefficients between IHF-NN, Volume and the other studied methods’ performance.

	IHF-NN	SVD	IHF-M	MOOD	GODIN	Vol	MCD	Ens	Ent
IHF-NN	1.00	0.38	**0.85**	0.23	−0.08	0.23	0.08	−0.08	−0.23
Volume	0.23	**0.54**	0.38	0.38	0.38	1.00	0.23	0.08	0.23

## Data Availability

The chest CT scans from the Mosmed-1110, MIDRC-RICORD-1a, MedSeg-9, MoscowCancer-500, LIDC/IDRI, LiTS, CT-ICH, VS-Seg, CrossMoDA, EGD and CC359 datasets used in this study are publicly available.

## Abbreviations

The following abbreviations are used in this manuscript:

AD	Anomaly detection
AUROC	Area under receiver operating characteristic
COVID-19	Coronavirus disease 19
CT	Computed Tomography
DL	Deep Learning
DSC	Dice similarity coefficient
FPR	False positive rate
ID	In-distribution
IHF	Intensity Histogram Features
MCD	Monte Carlo dropout
MDPI	Multidisciplinary Digital Publishing Institute
MOOD	Medical out-of-distribution
MRI	Magnetic resonance imaging
OOD	Out-of-distribution
PCA	Principal Component Analysis
SVD	Singular value decomposition
TPR	True positive rate
UE	Uncertainty estimation
